# SRR1 is essential to repress flowering in non-inductive conditions in *Arabidopsis thaliana*

**DOI:** 10.1093/jxb/eru317

**Published:** 2014-08-16

**Authors:** Mikael Johansson, Dorothee Staiger

**Affiliations:** Molecular Cell Physiology, Faculty for Biology, Bielefeld University, Bielefeld, Germany

**Keywords:** *Arabidopsis*, circadian clock, flowering time control, photoperiod, repressors, SRR1.

## Abstract

Timing of flowering is determined by environmental and developmental signals, leading to promotion or repression of key floral integrators. SENSITIVITY TO RED LIGHT REDUCED (SRR1) is a pioneer protein previously shown to be involved in regulation of the circadian clock and phytochrome B signalling in *Arabidopsis thaliana*. This report has examined the role of SRR1 in flowering time control. Loss-of-function *srr1-1* plants flowered very early compared with the wild type under short-day conditions and had a weak flowering response to increasing daylength. Furthermore, *FLOWERING LOCUS T* (*FT*) transcript levels were elevated already in short days in *srr1-1* compared with the wild type. This correlated with elevated end of day levels of *CONSTANS* (*CO*), whereas levels of *CYCLING DOF FACTOR 1* (*CDF1*), a repressor of *CO* transcription, were reduced. *srr1-1 gi-2* and *srr1-1 co-9* double mutants showed that SRR1 can also repress flowering independently of the photoperiodic pathway. *srr1-1* flowered consistently early between 16 °C and 27 °C, showing that SRR1 prevents premature flowering over a wide temperature range. SRR1 also promotes expression of the repressors *TEMPRANILLO 1* (*TEM1*) and *TEM2*. Consequently their targets in the gibberellin biosynthesis pathway were elevated in *srr1-1*. SRR1 is thus an important focal point of both photoperiodic and photoperiod-independent regulation of flowering. By stimulating expression of the *FT*-binding repressors *CDF1*, *TEM1* and *TEM2*, and *FLC*, flowering is inhibited in non-inductive conditions.

## Summary

SRR1 regulates flowering time in *Arabidopsis* by integrating photoperiodic and photoperiod-independent signals. By promoting expression of several repressors of *FT*, SRR1 represses flowering in non-inductive conditions.

## Introduction

Due to their sessile lifestyle, plants need to be able to adapt to their local environment. In particular, the transition from a vegetative to a reproductive state is carefully timed to maximize reproductive success. An intricate system of proteins that relay environmental and physiological stimuli forms a network of signalling pathways that converge at a small number of ‘floral pathway integrator genes’ including *FLOWERING LOCUS T* (*FT*) and *SUPPRESSOR OF OVEREXPRESSION OF CONSTANS 1* (*SOC1*) (Srikanth and Schmid, 2011). These in turn activate ‘floral meristem identity genes’ such as *APETALA 1* (*AP1*) and *LEAFY* to trigger formation of flowers (Abe *et al.*, 2005; Wigge, 2005).

*Arabidopsis thaliana* is a facultative long-day (LD) plant. Increasing daylength and temperature in spring promote flowering by antagonizing inhibitory effects of FLOWERING LOCUS C (FLC) (Amasino, 2010; Andrés and Coupland, 2012). The photoperiod is sensed in the leaves by an endogenous timekeeper, the circadian clock. The circadian clock consists of transcriptional feedback loops through which clock proteins generate their own 24h oscillations (McClung, 2011; Staiger *et al.*, 2013). In *Arabidopsis*, the core clock loop consists of two Myb transcription factors LATE ELONGATED HYPOCOTYL (LHY) and CIRCADIAN CLOCK ASSOCIATED 1 (CCA1) peaking at dawn, and TIMING OF CAB EXPRESSION1 (TOC1) peaking at dusk, that reciprocally repress each other (Wang *et al.*, 1997; Schaffer *et al.*, 1998; Strayer *et al.*, 2000; Alabadi *et al.*, 2002). Interlocked with this core loop is the morning-phased loop comprising *PSEUDO-RESPONSE REGULATOR* 7 (*PRR*7) and *PRR9* that are connected to CCA1 and LHY (Matsushika *et al.*, 2000; Locke *et al.*, 2006; Zeilinger *et al.*, 2006). Further, an evening-phased loop comprises GIGANTEA (GI), TOC1, and the evening complex components EARLY FLOWERING 3 (ELF3), ELF4, and LUX ARRHYTHMO (Fowler *et al.*, 1999; Kolmos *et al.*, 2009; Pokhilko *et al.*, 2010; Nusinow *et al.*, 2011; Herrero *et al.*, 2012), completing the basic structure of the circadian clock of interlocked central, morning, and evening loops. The circadian clock regulates the key component of the photoperiodic pathway, the zinc finger protein CONSTANS (CO) (Putterill *et al.*, 1995; Suarez-Lopez *et al.*, 2001). The *CO* mRNA undergoes circadian oscillations with a peak 8–10h after dawn in non-inductive short days (SDs). As the CO protein is degraded in darkness, it does not accumulate to significant levels in SDs. Under inductive LDs, the *CO* mRNA peaks 12–16h after dawn. The clock protein GI and FLAVIN KELCH F BOX 1 (FKF1), an F box ubiquitin ligase, that both peak 10–14h after dawn in LDs, undergo a light-dependent interaction. The GI–FKF1 complex promotes degradation of the CYCLING DOF FACTORS (CDFs) that repress *CO* promoter activity (Imaizumi *et al.*, 2005; Sawa *et al.*, 2007; Fornara *et al.*, 2009; Song *et al.*, 2012). In particular, CDF1 has been proven to repress *CO* promoter activity and also to repress *FT* activity by binding to the *FT* promoter in the morning (Song *et al.*, 2012). CDFs are thus important to prevent early-day accumulation of *CO* as well as *FT* and, by extension, premature flowering.

Accumulation of *CO* mRNA in the light phase of LDs allows CO protein to accumulate and stimulate *FT* transcription in the companion cells. FT protein then moves through the phloem to the shoot apex to induce flower formation (Corbesier *et al.*, 1996; Jaeger and Wigge, 2007; Mathieu *et al.*, 2007).

Photoperiodic flowering can also be triggered in a CO-independent manner, via GI regulation of miR172 processing (Jung *et al.*, 2007). Furthermore, GI can bind directly to *FT* (Sawa and Kay, 2011).

In addition to LDs, an extended period of cold enables *Arabidopsis* plants to flower. This vernalization response prevents inappropriate flowering during LDs in the autumn and instead promotes flowering in the spring (Alexandre and Hennig, 2008; Kim *et al.*, 2009). Vernalization leads to down-regulation of the key repressor FLC, a MADS domain transcription factor that binds to the promoters of *FT* in the leaf and *SOC1* in the apical meristem (Helliwell *et al.*, 2006; Searle *et al.*, 2006).

Apart from very low temperatures, moderate changes in ambient growth temperature influence floral transition (Blazquez *et al.*, 2003; Balasubramanian *et al.*, 2006; Lee *et al.*, 2007). A temperature rise by 4 °C accelerates flowering in non-inductive SDs to the same extent as extension of the photoperiod to LDs. Photoreceptors are believed to be part of this thermosensory pathway. For example, *phyB* mutants flower earlier than wild-type plants at 22 °C but not at 16 °C (Halliday *et al.*, 2003). A complex of the MADS domain transcription factor SHORT VEGETATE PHASE (SVP) and a splice isoform of the FLC-related MADS transcription factor FLOWERING LOCUS M (FLM), FLM-β, represses transcription of *FT* to prevent flowering at colder temperatures (Lee *et al.*, 2013; Pose *et al.*, 2013). At higher temperatures, *FLM-β* is down-regulated at the expense of the splice isoform *FLM-δ* that forms a complex with SVP that cannot interact with DNA (Pose *et al.*, 2013). Additionally, SVP is degraded at higher temperatures (Lee *et al.*, 2013). This combined regulation of *FLM* alternative splicing and SVP protein stability favours flowering at higher temperatures.

Furthermore, the phytohormone gibberellic acid (GA) is required for flowering in SDs, and external application of GA on SD-grown plants results in a LD-like flowering behaviour (Wilson *et al.*, 1992). The action of the GA pathway has long been thought to be largely masked by the photoperiodic pathway under LD conditions (Reeves and Coupland, 2001). More recently, GAs have been assigned a role in floral induction in response to inductive LDs through activation of *FT* transcription in leaves and of the *SQUAMOSA PROMOTER BINDING PROMOTER LIKE* genes in the shoot apical meristem (Porri *et al.*, 2012). GA signalling has also been linked to photoperiodic regulation via the TEMPRANILLO (TEM) transcription factors (Osnato *et al.*, 2012). TEM1 binds to a regulatory region in the first exon of the GA biosynthesis gene *GA3oxidase 1* (*GA3ox1*) to repress its expression and can thus control the amount of active GA in the plant and in this way influence flowering (Osnato *et al.*, 2012). Furthermore, the TEM1 and TEM2 proteins can directly repress *FT* to counteract CO activity, acting redundantly to each other (Castillejo and Pelaz, 2008; Osnato *et al.*, 2012)

A recent study combining genome-wide association and quantitative trait loci (QTL) mapping measured flowering time in ecologically realistic environmental conditions (Brachi *et al.*, 2010). In this field experiment, several genes associated with the circadian clock were identified, including *SENSITIVITY TO RED LIGHT REDUCED (SRR1)*. The loss-of-function *srr1* mutation has previously been shown to affect multiple outputs of the *Arabidopsis* circadian clock, including leaf movement rhythms and gene expression (Staiger *et al.*, 2003). The oscillations of morning- and evening-phased output genes as well as components of the core clock show a short period and reduced amplitude in *srr1*. *srr1* also exhibits reduced hypocotyl and petiole elongation in red light, showing that SRR1 is involved in phytochrome B (phyB) signalling. However, as circadian rhythms are affected in light–dark cycles, continuous light, and continuous darkness, SRR1 activity is probably required for normal clock function independently of a function in light input (Staiger *et al.*, 2003). Furthermore, *srr1* flowers early in a short photoperiod of 9h light. SRR1 is a pioneer protein whose sequence is very well conserved among a wide range of species, including mammals. Work performed in yeast showed that the *Saccharomyces cerevisiae* counterpart BER1 was involved in microtubule stability (Fiechter *et al.*, 2008), but its mode of action in plants is not known.

In this study, the role of SRR1 in flowering time control is characterized. The presented data show that SRR1 regulates expression of *CO*, *FT*, and *CDF1* in the photoperiodic pathway to inhibit flowering specifically in SDs. Furthermore, SRR1 connects the expression of the TEM1 and TEM2 transcription factors to the circadian clock. In addition, SRR1 can also repress flowering independently of photoperiod, demonstrated by the genetic relationships between SRR1 and CO and GI. SRR1 is thus acting as an integrator between photoperiodic regulation and other pathways to maintain repression of flowering in conditions not suitable for reproduction.

## Materials and methods

### Plant materials and growth conditions

The T-DNA mutant *srr1* in the Col-7 background has been described (Staiger *et al.*, 2003) and has now been renamed *srr1-1*. Additional *srr1* alleles from the SALK T-DNA collection (SALK_132099 and SALK_077868) have been characterized here and were named *srr1-2* and *srr1-3*, respectively. *co-9* was obtained from D. Weigel (Balasubramanian *et al.*, 2006). *gi-2* and *phyB-9* were acquired from the NASC stock centre. Mutations were confirmed using PCR, with the primers listed in Supplementary Table S1 available at *JXB* online.

All seeds were stratified for 3 d at 4 °C before put on soil. Seeds grown on plates were surface-sterilized and stratified for 3 d before they were sown on agar-solidified half-strength MS (Murashige and Skoog) medium (Duchefa) supplemented with 0.5% sucrose and 0.5g MES l^–1^.

For GA treatment, plants growing on soil were sprayed with 100 μM GA_3_ in the middle of the light period [Zeitgeber time (ZT) 4–6] once a week starting at day 10 after stratification. Mock treatment was performed by spraying with 0.1% dimethylformamide (DMF)/0.02% Tween-20. For paclobutrazol treatment, plants growing on soil were watered with 5 μM paclobutrazol in the middle of the light period (ZT4–6) once a week starting at day 10 after stratification. Mock treatment was performed by watering with 0.1% DMF/0.02% Tween-20. Vernalization treatment was performed as previously described (Streitner *et al.*, 2008). Plants were grown in Percival incubators AR66-L3 (CLF laboratories) in 150 μmol m^–2^ s^–1^ light intensity, with the light–dark and temperature conditions as indicated.

### Flowering experiments

Seeds were germinated as described above and grown on soil in a randomized fashion in Percival incubators AR66-L3 (CLF Laboratories). For ambient temperature flowering experiments, plants were grown at 16 °C or 20 °C before being shifted to 20 °C or 27 °C, respectively. Flowering time was determined by counting the rosette leaves once the bolt was 0.5cm tall (Steffen *et al.*, 2014). Mean values ±SD were calculated.

### Transcript analysis

Above-ground material, or leaves without the apex and apically enriched material, respectively, was harvested separately, as indicated, and immediately frozen in liquid N_2_. A green safe light was used for sampling in the dark periods. Samples were ground in a bead mill (Retsch MM400, www.retsch.com, last accessed 24 July 2014) using stainless steel beads. Total RNA was extracted using the Universal RNA kit (Roboklon, Berlin, Germany) and reverse transcribed. Quantitative PCR was performed as described (Streitner *et al.*, 2012) using the iTaq kit (Bio-Rad, www.bio-rad.com, last accessed 24 July 2014) on a Bio-Rad CFX-96 Realtime Detection System. C_T_ values were determined and relative expression levels were calculated based on non-equal efficiencies for each primer pair (Pfaffl, 2001). Data were normalized to *PP2A* (At1g13320) and expressed as the mean expression levels ±SE based on three biological replicates. Primers used are listed in Supplementary Table S1 at *JXB* online.

## Results

### SRR1 represses flowering in SDs

As flowering time control in response to the photoperiod depends on the circadian clock measuring the daylength, loss or misregulation of proteins involved in the circadian clock often results in a flowering phenotype (Schaffer *et al.*, 1998; Somers *et al.*, 1998; Wang and Tobin, 1998; Kim *et al.*, 2005). Similarly, the *srr1* T-DNA mutant shows impaired circadian rhythms and flowers very early in 9h light–15h dark cycles (Staiger *et al.*, 2003).

To characterize the photoperiodic response of *srr1* in detail, *srr1* and Col-7 wild-type (wt) plants were grown in photoperiods of different length. Flowering was accelerated with increasing daylength, with wt plants forming about half the number of rosette leaves in 12h light–12h dark compared with SDs (8h light–16h dark) and again forming about half the number of rosette leaves in LDs (16h light–8h dark) compared with 12h light–12h dark (Supplementary Fig. S1 at *JXB* online). In contrast, the acceleration of flowering in *srr1* in 12h light–12h dark compared with SDs was only moderate, and an additional extension by 4h to LDs resulted in only a small further acceleration. Thus, *srr1* responded much more weakly to increasing daylength than the wt.

To obtain independent confirmation of the flowering phenotype, additional T-DNA alleles from the SALK collection were characterized. The position of the T-DNA was confirmed using PCR, and homozygous lines were identified (Supplementary Fig. S2A at *JXB* online). The line SALK 132099, named *srr1-2*, has a T-DNA insertion in the promoter region of *SRR1*, 400bp upstream of the ATG. *SRR1* transcript levels in *srr1-2* were reduced to ~60% of the wt levels (Supplementary Fig. S2B). *srr1-2* flowered moderately earlier compared with the wt in SDs (Supplementary Fig. S2C). Another line, SALK 077868, named *srr1-3*, has a T-DNA insertion in the 5′-untranslated region, 271bp upstream of the ATG. *SRR1* transcript levels were unchanged and flowering was unaffected compared with the wt (Supplementary Fig. S2B, C). *srr1* in the Col-7 background, which does not express *SRR1* transcript at all (Staiger *et al.*, 2003) and showed the most pronounced early flowering phenotype, was renamed *srr1-1* and used in all subsequent experiments.

To show that the flowering phenotype of the mutant is caused by the loss of SRR1, *srr1-1* plants transformed with a construct where the *SRR1* coding sequence and a green fluorescent protein tag was expressed from the endogenous *SRR1* promoter (Staiger *et al.*, 2003) were assayed for flowering time. Independent transformants displayed wt-like flowering in both SDs and LDs (Supplementary Fig. S2D, E at *JXB* online). Thus, SRR1 complements the *srr1-1* flowering phenotype.

### SRR1 inhibits flowering through regulation of photoperiod components

**Fig. 1.**
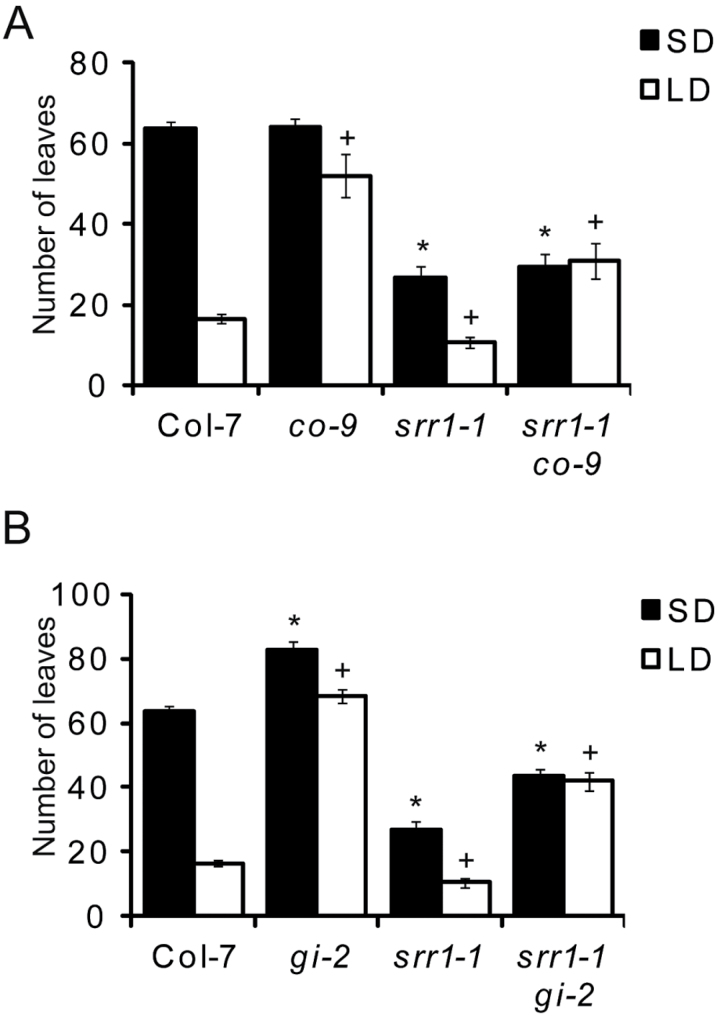
Flowering time of *srr1-1 co-9* and *srr1-1 gi-2* double mutants. Flowering time of Col-7 wt, *co-9*, *srr1-1* and *srr1-1 co-9* (A) and Col-7 wt, *gi-2*, *srr1-1* and *srr1-1 gi-2* (B) in SDs and LDs. Data represent means of rosette leaves ±SD (*n* >10). Statistical significance was tested using a two-tailed Student’s *t*-test. Asterisks indicate *P*-values of <0.01 between the wt and mutant in SDs. Crosses indicate *P*-values of <0.01 between the wt and mutant in LDs. Experiments were performed three times with similar results.

Since the response to increasing daylength in *srr1-1* plants was severely reduced, the functionality of the photoperiodic pathway in *srr1-1* was examined. To do this, the *srr1-1* mutation was introduced into the *co-9* mutant background by crossing. While the *co-9* mutation greatly delayed flowering in LDs (52±5.2 leaves), the *srr1-1 co-9* double mutant displayed an intermediate phenotype (30.9±4.5 leaves), flowering later than *srr1-1* (10.6±1.4 leaves) but earlier than *co-9* (Fig. 1A) (Student’s *t*-test, *P*<0.01). In SDs, the *co-9* mutant flowered in the same way as the wt (64±2.2 leaves versus 63.6±1.8 leaves), while the *srr1-1 co-9* double mutant flowered like the *srr1-1* single mutant (29.4±3.3 leaves versus 26.8±2.6 leaves) (Fig. 1A). No difference in leaf numbers was observed between SDs and LDs in the double mutant.

To test whether SRR1 affects the CO-independent branch of the photoperiodic pathway, double mutants with *gi-2* were also generated. The *gi-2* mutation strongly delayed flowering in LDs (68.5±2.2 leaves), while the *srr1-1 gi-2* double mutant displayed an intermediate phenotype (42±2.8 leaves), flowering later than *srr1-1* but earlier than *gi-2* (Fig. 1B). In SDs, the *srr1-1 gi-2* double mutant flowered somewhat later than *srr1-1* (43.6*±*2.3 leaves versus 26.8±2.6 leaves), but still earlier than both *gi-2* (82.9±2.3 leaves) and the wt (63.6±1.8 leaves) (Student’s *t*-test, *P*<0.01). Again there was no difference in leaf numbers between SDs and LDs for the *srr1-1 gi-2* double mutant. The intermediate flowering phenotypes in *srr1-1 co-9* and *srr1-1 gi-2* compared with the respective single mutants in LDs suggests that SRR1 has a dual mode of action to repress flowering, partly through the photoperiodic pathway but also in a photoperiod-independent manner. This is supported by the observation that a loss of SRR1 accelerates flowering in the *co-9* background in non-inductive SD conditions, where the photoperiodic pathway is not active.

**Fig. 2.**
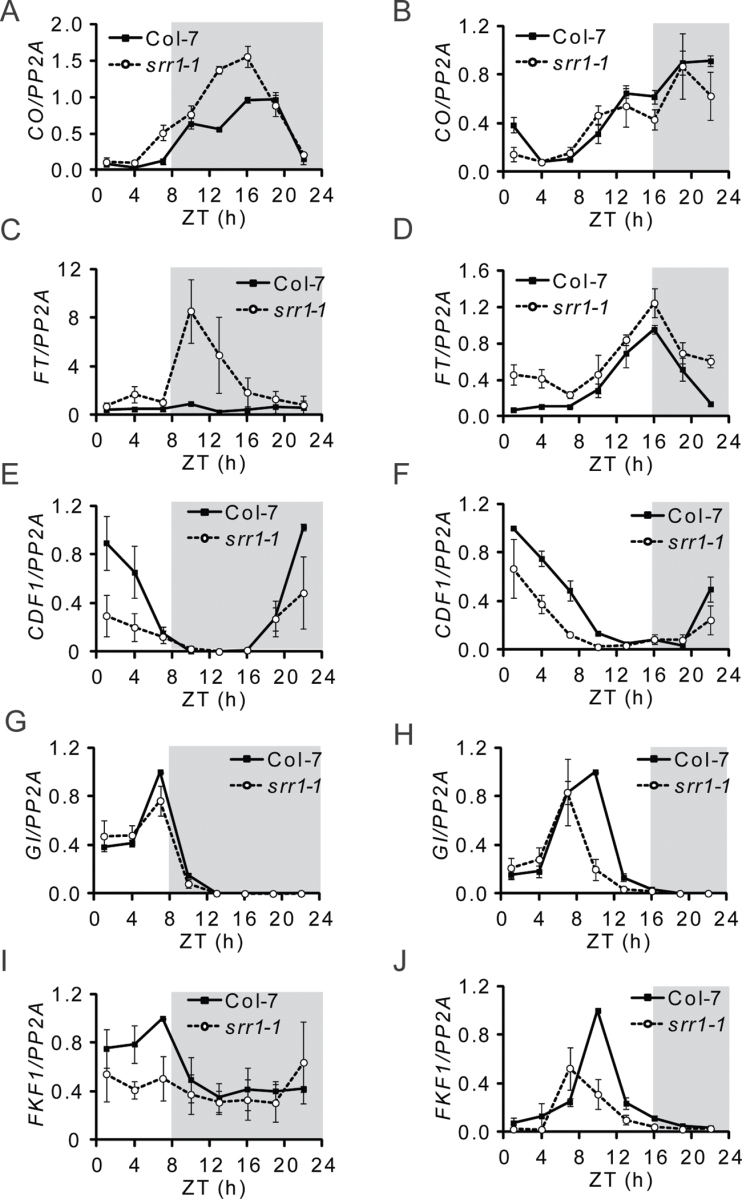
Expression of genes involved in photoperiodic regulation of flowering in *srr1-1*. Transcript levels of *CO (*A, B)*, FT* (C, D), *CDF1* (E, F)*, GI* (G, H), and *FKF1* (I, J) in SDs (A, C, E, G, I) and LDs (B, D, F, H, J) were determined by real-time PCR in 10-day-old seedlings. Samples were taken at 3h intervals starting at ZT1. Each data point is the average of three biological replicates ±SE. The grey fields represent the dark period.

To substantiate this behaviour further, transcript patterns of *CO* and *FT* were analysed in wt and *srr1-1* plants sampled every 3h, starting at ZT1 (1h after lights on), in SDs and LDs. *CO* levels were elevated in SDs in *srr1-1* compared with the wt, at the end of the light phase (ZT7), but not in LDs (Fig. 2A, B). Furthermore, *FT* levels, while low at all time points in the wt in SDs, were strongly elevated at the beginning of the dark phase, with a peak around ZT10 in *srr1-1* (Fig. 2C, D). *FT* thus displayed an LD-like transcript pattern, with the peak at the beginning of the dark period (ZT10 in SDs versus ZT16 in LDs). This strong and early accumulation of *FT* supports the flowering phenotype under non-inductive photoperiods. In LDs, *FT* levels were only moderately elevated in *srr1-1* compared with the wt. This could explain the moderate early flowering phenotype of *srr1-1* in LDs.

CDF1 is an important repressor of *CO* and *FT* expression during the morning (Imaizumi *et al.*, 2005; Sawa *et al.*, 2007; Fornara *et al.*, 2009). *CDF1* transcript levels were reduced in *srr1-1* compared with the wt at the end of the night and throughout the light phase in SDs (Fig. 2E, F). Thus, earlier accumulation of *CO* correlates with lower *CDF1* at a time when *CO* is repressed in the wt (Fig 2A, E).

GI and FKF1 form a complex that degrades CDF proteins in the second half of the light period, mainly in LDs (Song *et al.*, 2012). *GI* levels were largely unchanged in *srr1-1* compared with the wt in SDs, while a somewhat narrower peak of transcript accumulation could be seen in LDs (Fig. 2G, H). *FKF1* transcript levels were reduced in *srr1-1* in SDs between ZT1 and ZT7 and in LDs at ZT10 (Fig. 2I, J). A lower peak of *FKF1* expression could also be observed in LDs, similar to what could be seen for *GI* (Fig. 2H, J). Lower levels of FKF1 should hypothetically lead to higher CDF protein levels. However, since CDF degradation via FKF1 occurs later in the day, it may be of little significance for the *srr1-1* flowering phenotype in SDs, partly due to the already reduced *CDF1* transcript levels.

Thus, in *srr1-1*, both the reduction in *CDF1* peak transcript levels and the early increase in *CO* levels most probably contribute to the rhythmic *FT* transcript pattern seen in SDs, by reduced repression and increased activation of *FT*, respectively.

### *srr1-1* responds only weakly to vernalization

**Fig. 3.**
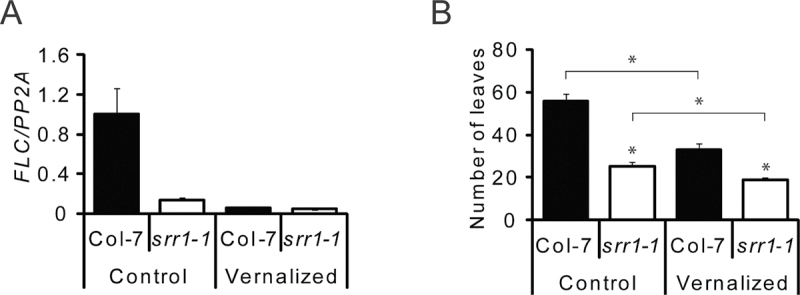
Vernalization response of *srr1-1. FLC* levels in Col-7 and *srr1-1* plants before and after vernalization determined using real-time PCR in seedlings with or without vernalization treatment (A). Each data point is the average of three biological replicates ±SE. Flowering time of Col-7 and *srr1-1* grown in SDs with and without vernalization (B). Data represent means of rosette leaves ±SD (*n* >10). Statistical significance was tested using a two-tailed Student’s *t*-test. Asterisks indicate *P*-values of <0.01 between the wt and mutant or between different treatments, as indicated by the bars. Experiments were performed twice with similar results.

*srr1-1* early flowering correlates with elevated levels of the floral integrator *FT* that is reciprocally regulated by CO and FLC. The *FLC* transcript level was strongly reduced in *srr1-1* compared with the wt (Fig. 3A). It was further reduced by vernalization to levels similar to those in vernalized wt plants. Accordingly, vernalized *srr1-1* plants flowered with fewer leaves than untreated plants, but the vernalization response was much weaker than in wt plants (Fig. 3B). Thus, even when the wt and *srr1-1* have comparable low levels of *FLC*, *srr1-1* flowers earlier than the wt. Furthermore, the low *FLC* level in *srr1-1* at 20 °C probably limits the effect of the vernalization treatment on flowering time in this mutant.

### *srr1-1* plants respond to ambient temperature changes

**Fig. 4.**
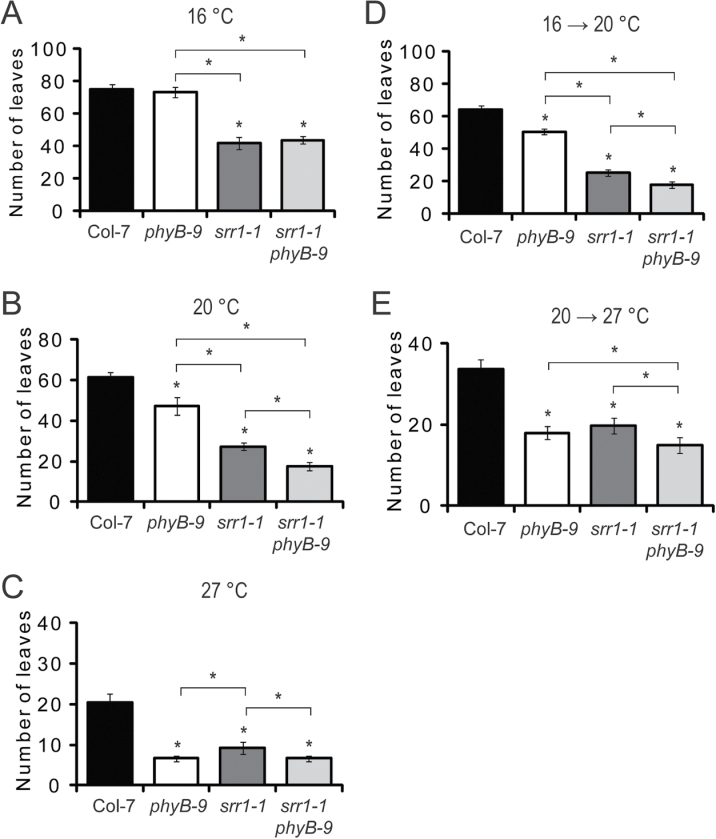
Temperature responses of *srr1-1* and *srr1-1 phyB-9.* Flowering of plants grown at a constant temperature of 16 °C (A), 20 °C (B), or 27 °C (C). Flowering of plants shifted from 16 °C to 20 °C (D) and from 20 °C to 27 °C (E). Temperature-shifted plants were grown at their initial temperature for 4 weeks, before being moved to a higher growth temperature. Data represent means of rosette leaves ±SD (*n* >10). Statistical significance was tested using a two-tailed Student’s *t*-test. Asteriks indicate *P*-values of <0.01 between the wt and mutant, or between different genotypes, as indicated by the bars. Experiments were performed three times with similar results.

An increase in ambient temperature accelerates flowering in *Arabidopsis* (Balasubramanian *et al.*, 2006). To examine the behaviour of *srr1-1* in different temperatures, plants were grown at 16, 20, and 27 °C. After 4 weeks, a subset of these plants was shifted from 16 °C to 20 °C and another subset from 20 °C to 27 °C. Both wt and *srr1-1* plants flowered earlier, with fewer leaves when grown at a constant temperature of 20 °C compared with 16 °C, and earlier when grown at a constant temperature of 27 °C compared with 20 °C (Fig. 4A–C). *srr1-1* consistently flowered earlier than the wt, with about half the number of leaves. A shift from 16 °C to 20 °C or from 20 °C to 27 °C promoted flowering in both wt and *srr1-1* plants, compared with the plants kept at constant 16 °C or 20 °C, respectively (Fig. 4A, B, D, E).

SRR1 has been implicated in phyB signalling and, because PhyB protein levels in *srr1-1* are similar to those in the wt, SRR1 probaby acts downstream of phyB (Staiger *et al.*, 2003). *phyB* mutants lose their early flowering phenotype when grown at 16 °C (Halliday *et al.*, 2003). To investigate whether SRR1 mediates *phyB* signals to control flowering, a *srr1-1 phyB-9* double mutant was generated and grown at different ambient temperatures. At 16 °C, where mutations in *phyB* have no effect on flowering, the *srr1-1 phyB-9* double mutant flowered with the same number of leaves as the *srr1-1* mutant (Fig. 4A). At 20 °C the effect of the two mutations was additive, with the double mutant flowering earlier than the *srr1-1* mutant (Fig. 4B). In plants grown at 27 °C, both the *phyB*-*9* and *srr1-1 phyB-9* mutants responded very strongly to the high temperature by flowering with only ~6.7±0.7 leaves and earlier than *srr1-1* (9.2±1.4 leaves) (Fig. 4C). The *srr1-1 phyB-9* plants shifted from 16 °C to 20 °C or from 20 °C to 27 °C also responded with accelerated flowering, with an additive phenotype compared with *srr1-1* (Fig. 4D, E). In conclusion, SRR1 is not affected by lack of phyB at 16 °C, while at 20 °C both proteins contribute to repression of flowering, and at 27 °C SRR1 seems to depend on phyB for its control of flowering.

### Regulation of flowering time components by SRR1

To identify downstream targets of SRR1 in flowering time control, the expression of known flowering time genes was compared in *srr1-1* and wt plants under non-inductive and inductive conditions. Plants were grown in SD conditions at 20 °C for 3 weeks and subsequently shifted to 27 °C for 5 d. Leaf material and apically enriched material was harvested separately in the second half of the photoperiod (ZT6).

**Fig. 5.**
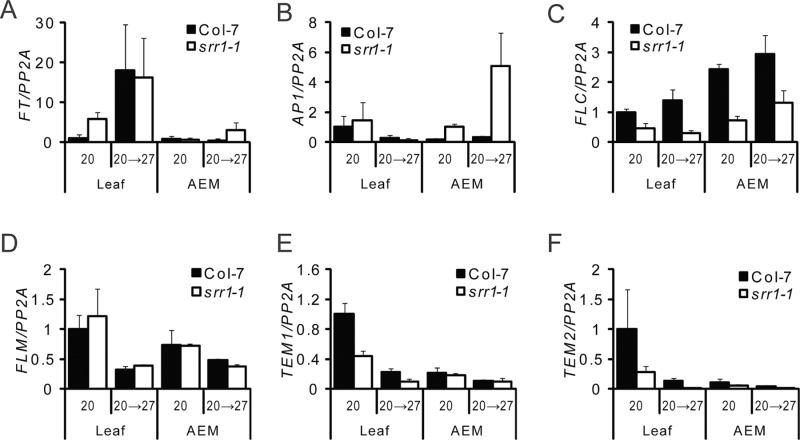
Transcript analysis of flowering time genes. Plants were grown for 3 weeks at 20 °C in SDs and a subset were subsequently shifted to 27 °C for 5 d before sampling. *FT* (A), *AP1* (B), *FLC* (C), *FLM* (D), *TEM1* (E), and *TEM2* (F) transcript levels were determined using real-time PCR. Expression levels are relative to *PP2A*. Shown is the mean expression based on three biological replicates each ±SE. AEM, apically enriched material.

The *FT* level was higher in leaves of *srr1-1* plants compared with wt plants under non-inductive conditions (Fig. 5A). Upon transfer to 27 °C, *FT* increased, reaching similar levels in wt plants and *srr1-1* plants. The weak signal in apically enriched material probably reflects expression in residual leaf material. In concert with elevated *FT* levels in the leaf, the meristem identity gene *AP1* was more strongly expressed in the apically enriched material of *srr1-1* compared with the wt at 20 °C. *AP1* was strongly induced at 27 °C and showed a higher level of expression in *srr1-1* compared with the wt (Fig. 5B). The increased levels of *FT* in the leaves in *srr1-1* under non-inductive growth conditions correlate with the early flowering phenotype in SDs, and the response to an inductive treatment is consistent with the weaker flowering phenotype in *srr1-1* under inductive conditions.

The floral integrator gene *SOC1* was expressed at somewhat higher levels in *srr1-1* compared with the wt at 20 °C in the leaves (Supplementary Fig. S3A at *JXB* online). Transcript levels of *SOC1* decreased somewhat after the shift to 27 °C in leaves, but expression levels were too low to draw any conclusions about changes in apically enriched material, where SOC1 activity is important for flowering. FLC acts as a repressor of flowering by binding to the *FT* promoter in the leaves and repressing *FD* and *SOC1* in the shoot apical meristem (Searle *et al.*, 2006). In *srr1-1*, *FLC* levels were decreased in both leaves and apically enriched material compared with wt plants, which probably contributes to early flowering of *srr1-1* (Figs 3B, 5C). Little difference could be seen in *FLC* levels upon transfer to 27 °C, similar to earlier findings (Edwards *et al.*, 2006). FLC has been shown to have a role in suppressing thermal induction in ecotypes with high *FLC* levels (Balasubramanian *et al.*, 2006), but it has also been noted that FLC is not a major player in ambient temperature-responsive flowering in the Col ecotype (Blazquez *et al.*, 2003).

The levels of *SVP*, a key component of the ambient temperature pathway, were similar in *srr1-1* and the wt (Supplementary Fig. S3B at *JXB* online). *FLM* transcript levels decreased in response to the increased temperature in both *srr1-1* and the wt (Fig. 5D). Ratios between the repressive *FLM-β* isoform and the competing *FLM-δ* isoform decreased from ~5 at 20 °C to ~2 at 27 °C in the leaves and from ~4 at 20 °C to ~2 in 27 °C in the apically enriched material, in both *srr1-1* and the wt (Supplementary Fig. S3C–E). This is similar to what has previously been reported (Pose *et al.*, 2013). Since no differences were observed between *srr1-1* and the wt, the temperature response pathway was not consistently altered in *srr1-1*.

The transcription factors TEM1 and TEM2 are direct repressors of *FT* and have been shown to antagonize CO activation of *FT* in a redundant manner (Castillejo and Pelaz, 2008). Moreover, they have recently been shown to establish and control the length of juvenility and also repress *CO* expression (Sgamma *et al.*, 2014). Down-regulation of both *TEM1* and *TEM2* expression is necessary for a plant’s ability to respond to inductive photoperiods, through accumulation of *FT*. Both *TEM1* and *TEM2* were expressed at reduced levels in leaves of *srr1-1* compared with the wt at 20 °C (Fig. 5E, F). Upon transfer to 27 °C, *TEM1* and *TEM2* levels were strongly reduced in the wt and further reduced in *srr1-1*, in correlation with derepression of flowering in response to an ambient temperature increase. In the apically enriched material, only very low expression was detected, which was not significantly different between the wt and *srr1-1* or between 20 °C and 27 °C. Expression of *TEM1* and *TEM2* transcripts is thus temperature sensitive.

The *SRR1* transcript itself was expressed in both leaves and apically enriched material. It was not up-regulated by increased ambient temperature (Supplementary Fig. S3F at *JXB* online). Thus, *SRR1* does not respond to increases in temperature.

### GA biosynthesis components are changed in *srr1-1*

**Fig. 6.**
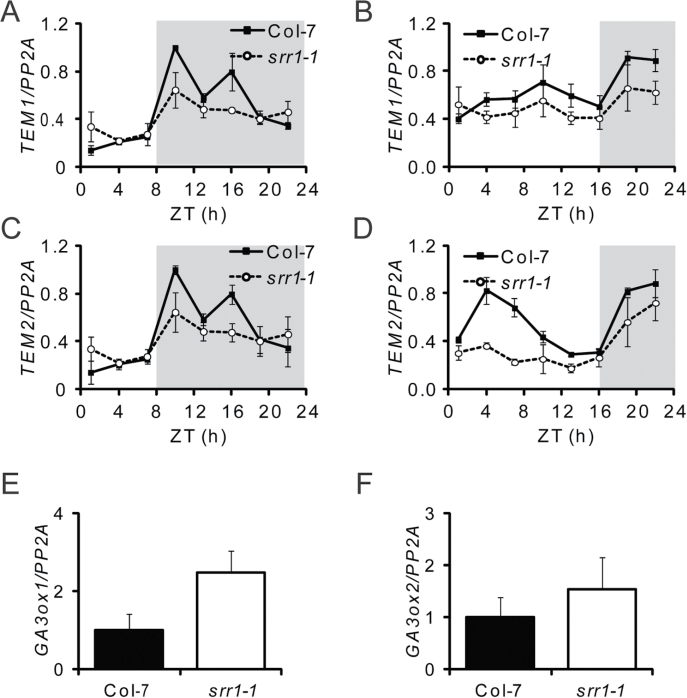
Transcript analysis of *TEM1*, *TEM2*, and GA biosynthesis components. Expression of *TEM1* (A, B) and *TEM2* (C, D) in SDs (A, C) and LDs (B, D) in 10-day-old seedlings. Samples were taken every 3h starting at ZT1. The grey fields represent the dark period. Expression of *GA3ox1* (E) and *GA3ox2* (F) in plants grown at 20 °C and plants treated with a 27 °C temperature increase. Transcript levels were determined by real-time PCR. Each data point is the average of three biological replicates ±SE. Expression levels are relative to *PP2A*.

To further examine the behaviour of *TEM1* and *TEM2* in *srr1-1*, their expression was tested in *srr1-1* and the wt throughout the day. Both *TEM1* and *TEM2* showed peaks after dusk in SDs and LDs (Fig. 6A–D), correlating with a previous report (Osnato *et al.*, 2012). The *srr1-1* mutation led to somewhat lower levels of *TEM1* and *TEM2* in SDs (Fig. 6A, C) and LDs (Fig. 6B, D). The peak of *TEM1* and *TEM2* in SDs around ZT12 has been proposed to be important for repression of *FT* in SDs (Osnato *et al.*, 2012), suggesting that lower *TEM1* and *TEM2* expression contributes to derepression of *FT* in *srr1-1*.

TEM1 and TEM2 have also been shown to regulate GA metabolism in *Arabidopsis* in concert with photoperiod (Castillejo and Pelaz, 2008; Osnato *et al.*, 2012). TEM1 directly represses the expression of the GA biosynthetic genes *GA3oxidase1* (*GA3ox1*) and *GA3ox2* (Osnato *et al.*, 2012). To examine whether the effect of the *srr1-1* mutation on *TEM1* and *TEM2* transcript levels also affected expression of genes involved in GA biosynthesis, transcript levels of *GA3ox1* and *GA3ox2* were tested in plants grown at 20 °C in SDs. *GA3ox1* levels were elevated in *srr1-1* compared with the wt, while no difference could be observed in *GA3ox2* levels (Fig. 6E, F). Previously, a higher up-regulation of *GA3ox1* expression than of *GA3ox2* was observed in *tem1-1 tem2-1* loss-of-function mutants (Osnato *et al.*, 2012). The smaller effect in the *srr1-1* mutant, compared with *tem1-1 tem2-1*, is most probably due to the fact that *TEM1* and *TEM2* are still expressed in *srr1-1*, although at lower levels. Also the level of the GA biosynthesis gene *GA20ox2*, which is down-regulated in response to overexpression of TEM1, was somewhat higher in *srr1-1* (Supplementary Fig. S4A at *JXB* online). The catabolic enzyme *GA2ox2* was unchanged between *srr1-1* and the wt, suggesting that deactivation of GA is unaffected (Supplementary Fig. S4B). Thus, through regulation of TEM1 and TEM2, SRR1 can affect *FT* repression and GA biosynthesis, which in both cases influence flowering.

To examine whether the overall GA response in *srr1-1* was affected, *srr1-1* and wt plants grown in SDs were treated with the bioactive GA_3_. This strongly promoted flowering in the wt (Supplementary Fig. S4C at *JXB* online). *srr1-1* plants reacted almost as strongly to exogenous GA_3_ as wt plants, flowering with about half the leaves of untreated *srr1-1* plants. This suggests that the GA pathway is functional in *srr1-1*. Treatment of SD-grown and LD-grown plants with the GA biosynthesis inhibitor paclobutrazol delayed flowering in both the wt and *srr1-1*, in line with the importance of GA also in LD conditions (Porri *et al.*, 2012). *srr1-1* still flowered earlier than the wt (Supplementary Fig. S4D, E).

## Discussion

Flowering in *Arabidopsis* is triggered by environmental factors such as increasing daylength (Andrés and Coupland, 2012) and temperature (Blazquez *et al.*, 2003). It is, however, not only important for plants to respond to environmental changes that are suitable for flowering. Equally important is the ability to accumulate sufficient resources before the transition to reproductive growth. Floral repressors have an important role in this as safeguards against premature transition to flowering (Yant *et al.*, 2009). *Srr1-1* plants flower very early in SDs compared with the wt. Lengthening of the photoperiod greatly advances floral transition in the wt but has only a small promotive effect in *srr1-1* (Supplementary Fig. S1 at *JXB* online), suggesting that SRR1 is more important in non-inductive conditions.

### SRR1 can affect flowering in ways both dependent on and independent of the photoperiodic pathway

The clock-controlled *CO* transcript oscillation with a peak in the dark determines the flowering response to LDs (Suarez-Lopez *et al.*, 2001; Valverde *et al.*, 2004). In *srr1-1* plants, *CO* transcript levels were increased and *CO* started to accumulate already during the light period in SDs, possibly due to an advanced phase resulting from the *srr1-1* clock phenoype (Fig. 2A). This correlated with lower peak transcript levels of the *CO* repressor *CDF1*, compared with the wt (Fig. 2E, F). Thus, lower CDF levels most probably result in higher CO protein levels, which can promote *FT* expression. In addition, CDF1 can directly repress *FT*, and the lower *CDF1* levels probably lead to derepression of *FT*.

As a result of these changes in *CO* and *CDF1* transcript levels, the expression of *FT*, which normally is repressed at all time points in non-inductive conditions, has an LD-like pattern in *srr1-1* in SDs, with a peak of expression at the beginning of the dark period. It thus seems as if the *srr1-1* mutation unmasks an underlying rhythm of *FT* expression, showing that SRR1 has an important role as an inhibitor of flowering in non-inductive conditions, ensuring that the photoperiodic response is not triggered.

Despite this, the introduction of *srr1-1* into the photoperiodic mutants *gi-2* and *co-9* resulted in accelerated flowering compared with *gi-2* and *co-9* single mutants, respectively, but delayed flowering compared wiht the *srr1-1* single mutant in LDs (Fig. 1A, B). In SDs, the *srr1-1 co-9* double mutant flowered in the same way as *srr1-1* in SDs (Fig. 1A). Interestingly, both the *srr1-1 gi-2* and *srr1-1 co-9* double mutants flowered with the same number of leaves in SDs as in LDs, suggesting that the moderately earlier flowering phenotype of *srr1-1* plants in LDs is dependent on the photoperiodic pathway. This is most probably a result of decreased repression in the absence of SRR1 and less promotion of flowering throughout the photoperiodic pathway, rendering the double mutants photoperiod independent. The accelerated flowering by *srr1-1* in the *co-9* and *gi-2* background in both SDs and LDs does however suggest that SRR1 clearly can act independently of the photoperiod to regulate flowering. Thus, SRR1 can repress flowering in a dual mode, both through the photoperiodic pathway that is controlled by the circadian clock and in a photoperiod-independent manner.

### SRR1 regulates several transcription factors that are repressors of *FT*

*FT* levels were higher in *srr1-1* compared with the wt at 20 °C and increased in response to a flowering-inducing temperature shift from 20 °C to 27 °C, in both *srr1-1* and the wt (Fig. 5A). This confirmed that *FT* is derepressed under non-inductive conditions in *srr1-1*.

The decreased transcript levels of the *FT* repressors *TEM1* and *TEM2* in *srr1-1* compared with the wt (Fig. 5E, F) and their diurnal expression profiles (Fig. 6A–D) with a lower peak of expression in the dark phase in SDs explains part of the derepression of *FT*. Partial suppression of *TEM1* and *TEM2* may not fully explain the early flowering of *srr1-1*, due to redundancy of the single *tem1* and *tem2* mutants; however, as both *TEM1* and *TEM2* expression is reduced in *srr1-1*, an effect on *FT* levels is likely.

The peaks of *TEM1* and *TEM2* in the dark phase have been proposed to be important not only for *FT* repression but also for regulation of GA biosynthesis components (Osnato *et al.*, 2012). Consequently, the TEM1/TEM2 targets in the GA biosynthesis pathway, *GA3ox1* and *GA3ox2*, were somewhat increased (Fig. 6E, F).

Lower *TEM1* and *TEM2* levels in *srr1-1* led to less repression of *FT* and thus accelerated flowering and indirect (positive) effects on flowering through increased activity in the GA biosynthesis pathway. In addition, this further connects the circadian clock and TEM1 and TEM2, where SRR1 helps to maintain *TEM1* and *TEM2* levels and in this way inhibits *FT* accumulation and flowering. GI has also been shown to interact with TEM1 and TEM2 on the protein level (Sawa and Kay, 2011). *GI* levels are, however, unchanged in the *srr1-1* background in SDs. A possibility is that SRR1 promotes *TEM1* and *TEM2* expression and that GI in turn interacts with the TEM1 and TEM2 proteins to regulate them. TEM1 and TEM2 are reported to counteract CO promotion of *FT* in a developmental manner, decreasing with increasing age of the plants, and also repress *CO* expression (Castillejo and Pelaz, 2008; Sgamma *et al.*, 2014). Since SRR1 can influence the transcript patterns of both CO and TEM1 and TEM2, it possibly acts to balance the expression between CO on the one hand and TEM1 and TEM2 on the other hand to sustain vegetative growth until both environmental and developmental factors favour transition to flowering.

### SRR1 represses flowering over a wide range of temperatures

s*rr1-1* flowered earlier than the wt under all tested temperatures, and responded to increases in temperature by accelerated flowering, showing that the temperature response in *srr1-1* is functional (Fig. 4). The *srr1-1 phyB-9* double mutant behaved like *srr1-1* at 16 °C, where the *phyB-9* mutation has no effect on flowering, while the effect of the two mutations was additive at 20 °C (Fig. 4A, B). At 27 °C, the double mutant flowered like *phyB-9*, with a very strong flowering response to the temperature. The consistent early flowering of *srr1-1* at all tested temperatures shows that SRR1 is necessary to prevent premature flowering in a wide temperature range. Moreover, the changing impact of the *phyB-9* mutation on the *srr1-1* flowering phenotype in different temperatures, ranging from no effect on the flowering phenotype of the *srr1-1 phyB-9* mutant at 16 °C to an additive effect at 20 °C and *phyB-9-*like flowering at 27 °C, suggests that the relationship between phyB and SRR1 could be temperature dependent (Fig. 4).

Furthermore, *srr1-1* plants showed a much weaker vernalization response than wt plants, probably because *FLC* transcript, encoding a key floral repressor, is already much lower in *srr1-1* than in wt plants before vernalization (Fig. 3). SRR1 can thus prevent flowering in a photoperiod-independent manner by promoting *FLC* expression.

### SRR1 integrates photoperiod-dependent and photoperiod-independent information to repress flowering in non-inductive conditions

**Fig. 7.**
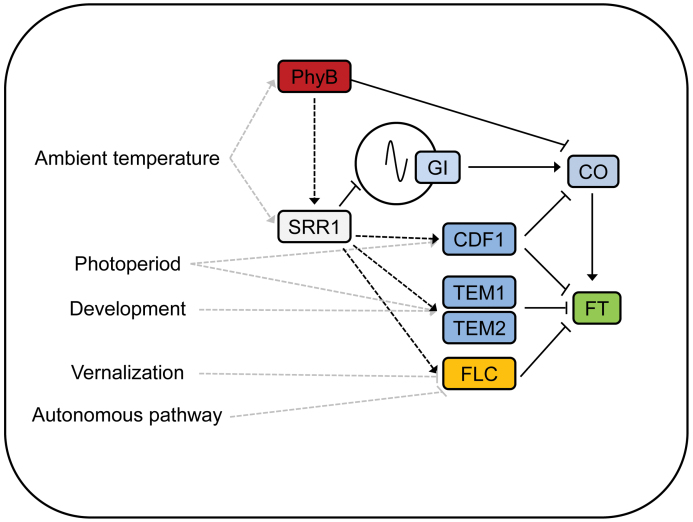
Conceptual model of SRR1 function. SRR1 has a function in setting the period of the circadian clock, as well as in phyB signalling. Expression of *FT* repressors involved in photoperiodic regulation of flowering—*CDF1*, *TEM1*, and *TEM2—*as well as the photoperiod-independent *FLC* is promoted by SRR1. *TEM1* and *TEM2* are also connected to developmental control of flowering, since their expression levels decrease with age. In this way, SRR1 prevents premature flowering in non-inductive SDs. In the *srr1-1* mutant, decreased repression of *FT* and early accumulation of *CO* leads to an LD-like expression pattern of *FT* and early flowering, especially in non-inductive environmental conditions. (This figure is available in colour at *JXB* online.)

The presented data reveal that SRR1 affects expression of several repressors of *FT*. Among those are transcription factors of different classes including the MADS domain protein FLC, the RAV (RELATED TO ABI3/VP1) family TEM1 and TEM2, and the Dof (DNA-binding with one finger) protein CDF1. This includes genes with no rhythmic expression (*FLC*), and rhythmic genes with an expression peak in the morning (*CDF1*) as well as with an expression peak in the dark (*TEM1*/*TEM2*). A working model for SRR1’s role in flowering time control is described in Fig. 7.

With *srr1-1* responding to all tested flowering-promoting treatments, the role of SRR1 seems not to be restricted to a specific signalling pathway, but rather to maintaining a basal level of repressive elements in non-inductive conditions. The early flowering phenotype of *srr1-1* plants in SDs thus seems to be the result of the combined effect of loss of expression of several transcription factors that act as direct repressors of *FT* expression, leading to an LD-like expression pattern of *FT*. Under inductive conditions such as LDs, other activating factors overcome the effect of SRR1 to trigger a flowering response. This could explain why SRR1 was identified as an important regulator of flowering in a genome-wide association and QTL mapping study on a plant population grown in field conditions over two seasons (Brachi *et al.*, 2010), since screens for flowering time regulators in laboratory conditions have been performed in conditions optimized for flowering that seldom occur in a realistic environment. Further, an SRR1 homologue in *Brassica rapa* was recently shown to be associated with flowering time control in a study combining flowering QTL analysis and whole-genome transcript variation (Xiao *et al.*, 2013). SRR1 appears to be a focal point of several pathways, necessary to synchronize photoperiodic regulation with other factors to maintain vegetative growth under non-inductive conditions. Thus, SRR1 is an upstream regulator of reproduction, preventing flowering until other factors signal that the time is suitable to shift from vegetative to reproductive growth.

## Supplementary data

Supplementary data are available at *JXB* online.

Figure S1. Flowering time of *srr1* in different photoperiods.

Fgure S2. Characterization of T-DNA insertion lines in *SRR1*.

Figure S3. Transcript analysis of *SVP*, *FLM-β*, *FLM-δ*, *SOC1*, and *SRR1*.

Figure S4. Transcript analysis of *GA20ox2* (A) and *GA2ox2* (B) in plants grown at 20 °C and subsequently shifted to 27 °C.

Table S1. List of primers used in this study.

Supplementary Data
