# Supplementary material for: SRR1 is essential to repress flowering in non-inductive conditions in Arabidopsis thaliana
Source: J Exp Bot. 2014 Aug 16;65(20):5811–22. doi: 10.1093/jxb/eru317 (PMC4203120; doi:10.1093/jxb/eru317)
Supplement: Supplementary Data [file supp_65_20_5811__index.html]

SRR1 is essential to repress flowering in non-inductive conditions in Arabidopsis thaliana — SRR1 is essential to repress flowering in non-inductive conditions in Arabidopsis thaliana — Supplementary Data 

# SRR1 is essential to repress flowering in non-inductive conditions in *Arabidopsis thaliana*

## Supplementary Data

Data files

**Files in this Data Supplement:**

- Supplementary Data - Supplementary Data
- Supplementary Data - Supplementary Data
