# Supplementary material for: SRR1 is essential to repress flowering in non-inductive conditions in Arabidopsis thaliana
Source: J Exp Bot. 2014 Aug 16;65(20):5811–22. doi: 10.1093/jxb/eru317 (PMC4203120; doi:10.1093/jxb/eru317)
Supplement: Supplementary Data [file supp_eru317_jexbot126045_file001.pdf]

Supplementary figure 1

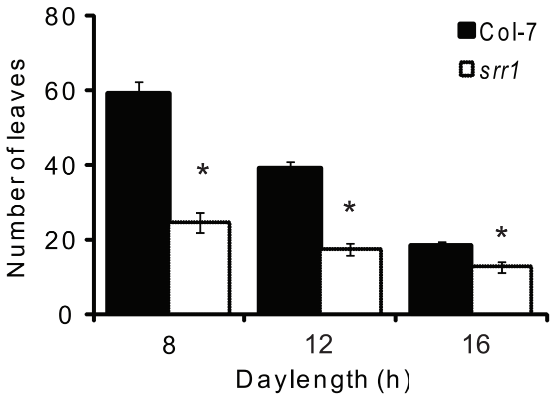

Supplementary figure 1 Flowering time of *srr1* in different photoperiods. Flowering time of Col-7 wt and *srr1* was measured in SDs (8 h L:16 h D), 12 h L-12 h D cycles and LDs (16 h L:8 h D), respectively. Rosette leaf number once the bolt was 0.5 cm. Data represent means + s.d. (n >10). Statistical significance was tested using a two-tailed Student's t-test. Stars indicate P-values of <0.01 between wt and mutant.

Supplementary figure 2

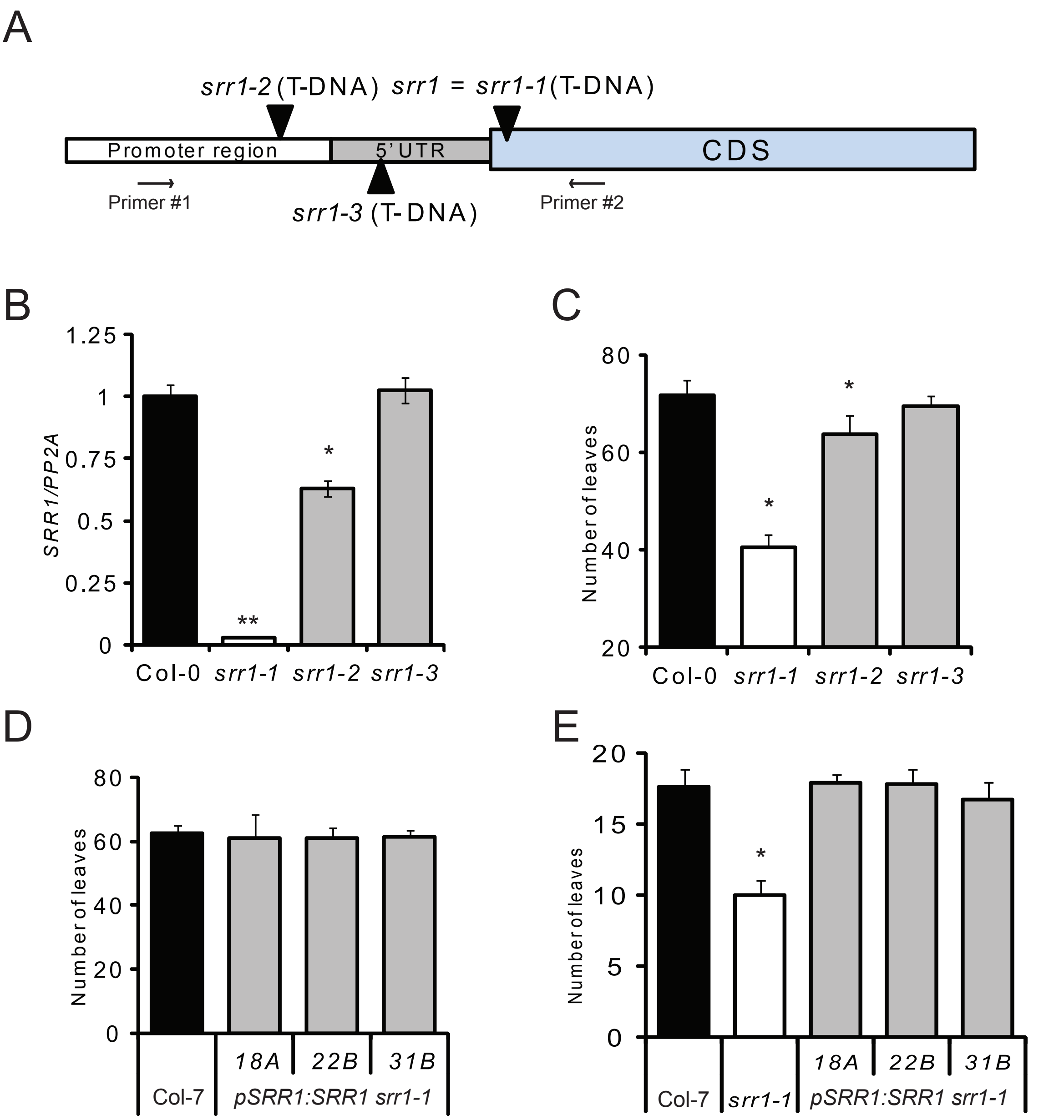

Supplementary figure 2 Characterization of T-DNA insertion lines in *SRR1*. (A) Schematic representation of the *SRR1* locus in relation to the T-DNA (represented as a triangle) in *srr1-1* and the SALK lines SALK 132093 (*srr1*-2) and SALK 077868 (*srr1*-3). The blue box depicts the coding sequence (CDS). PCR with primer set #1/ #2, which was used to amplify wild type Col-0 DNA, and primer set #1 / LBb1, which was used to amplify a junctionfragment containing both *A. thaliana* DNA and T-DNA confirmed that the *srr1-1, srr1-2* and *srr1-3* mutants are homozygous for the T-DNA insert (see Supplementary table 1). Expression of *SRR1* in the *srr1* alleles (B). Transcript levels were determined by Realtime PCR. Flowering in SDs of the *srr1* alleles (C). Flowering time in SDs (D) and LDs (E) of *srr1-1* lines complemented with *pSRR1:SRR1-GFP*. Statistical significance was tested using a two-tailed Student's t-test. Stars indicate P-values <0.01 between wt and mutant. Double stars indicate P-values <0.001 between wt and mutant. Experiments were performed twice with similar results.

Supplementary figure 3

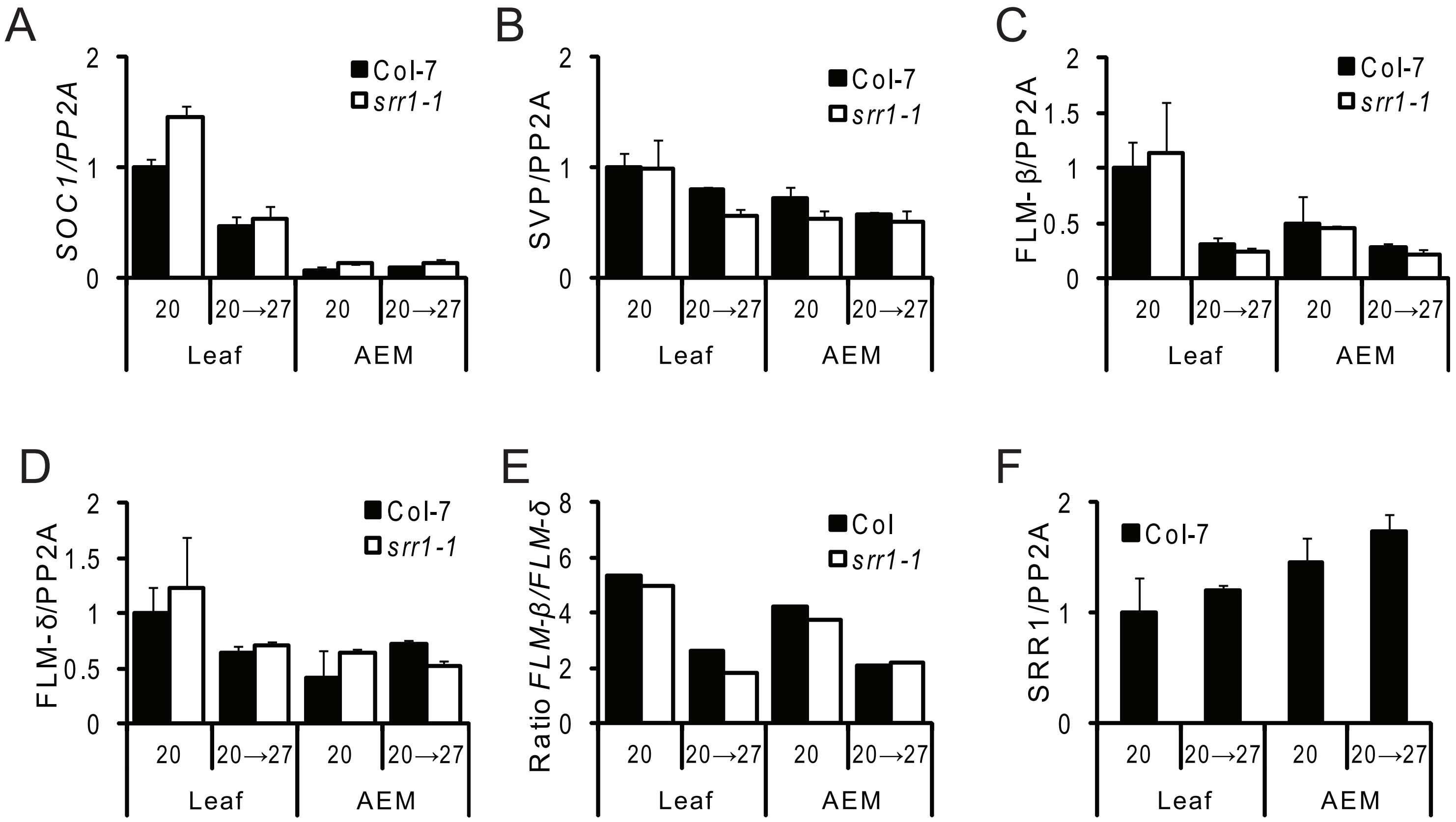

Supplementary figure 3 Transcript analysis of *SVP*, *FLM-β*, *FLM-δ*, *SOC1* and *SRR1*. Expression of *SVP* (A), *FLM-β* (B), *FLM-δ* (C), *SOC1* (D) and *SRR1* (E) in plants grown at 20 °C and plants treated with a 27 °C temperature increase. Transcript levels were determined by Realtime PCR. Each time point is the average of three biological replicates ± s. e. Expression levels are relative to *PP2A*. AEM = Apically enriched material.

Supplementary figure 4

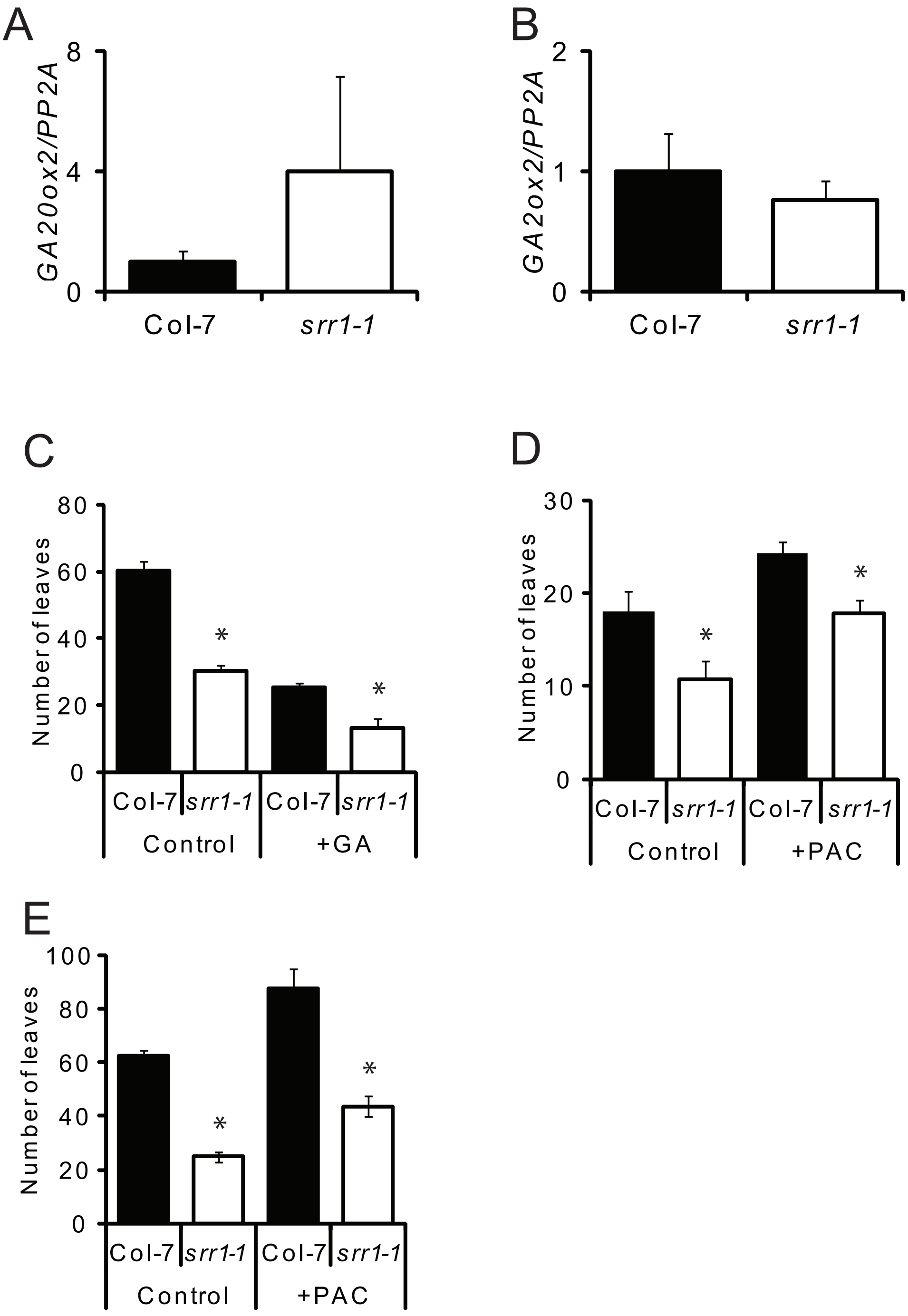

Supplementary figure 4 Transcript analysis of *GA20ox2* (A) and *GA2ox2* (B) in plants grown at 20 °C and subsequently shifted to 27 °C. Each datapoint is the average of three biological replicates ± s. e. Expression levels are relative to *PP2A*. AEM = Apically enriched material.

Flowering time of *srr1-1* in response to GA and paclobutrazol treatments. Plants treated with GA in SDs (C) and paclobutrazol in LDs (D) and SDs (E). Statistical significance was tested using a two-tailed Student's t-test. Stars indicate P-values of <0.01 between wt and mutant. Experiments were performed twice with similar results.
